# Special Issue: Applications of CRISPR Technology in Virology 2018

**DOI:** 10.3390/v11090839

**Published:** 2019-09-10

**Authors:** Dong-Yan Jin

**Affiliations:** School of Biomedical Sciences, The University of Hong Kong, 21 Sassoon Road, Pokfulam, Hong Kong; dyjin@hku.hk

Precision genome engineering by CRISPR is a game-changing technology that originates from the study of virus–host interaction and promises to revolutionize virology and antiviral therapy. CRISPR-mediated knock-in and knock-out of viral and cellular genes has been most revealing in the study of viral pathogenesis. CRISPR-based genome-wide forward genetic screens, viral delivery systems for CRISPR and other CRISPR-based enabling tools including those in live cell imaging, viral disease modeling and treatment have also come of age. The applications of CRISPR technology in virology are far-reaching. The CRISPR revolution will reach beyond the research lab. In this Special Issue of *Viruses*, we have assembled a collection of reviews and research papers focusing on applications of CRISPR technology in virology. This collection provides a glimpse of the impact of CRISPR technologies on various fields of virology such as viral genetics; the study of viral entry, virus–host interaction, viral pathogenesis and cellular response to viral infection; viral immunology; viral disease modeling; and design and development of vaccines and antivirals. Topics of special interest include viral delivery systems for CRISPR reviewed by Tsang and coworkers [1]; functional screening for host restriction and dependency factors using CRISPR reviewed by Gewurz and coworkers [2]; antiviral CRISPR targeting host and viral genes reviewed by Guo and coworkers [3] as well as Liu and coworkers [4], respectively; CRISPR in stem cells and organoids reviewed by Yoon and coworkers [5]; and CRISPR editing as a tool for viral reverse genetics and rapid genome cloning as reviewed by Moens and coworkers for orthopoxviruses [6], by Kanda and coworkers for Epstein-Barr virus [7], and by Hatoum-Aslan for phages [8]. In addition, there are six research articles that shed light on the use of CRISPR to create models for the study of viral infection by Wang et al. [9]; CRISPR-based mutant virus construction by Banfield et al. [10]; bioinformatic tools for CRISPR typing and analysis of CRISPR loci by Moineau et al. [11]; genome editing of an archaeal virus based on CRIPSR and anti-CRISPR by Peng et al. [12]; engineering RNA virus interference in *Arabidopsis* using CRISPR/Cas13 by Mahfouz et al. [13]; and the impact of viral genetic diversity on CRISPR antiviral activity and viral escape by Das et al. [14]. It is hoped that this Special Issue containing 14 papers not only provides an update on the emerging field but also serves as a reference and guide to virologists who are and will be using CRISPR in their research.

As in most cases, development of CRISPR technology is the result of blue skies research. The success story of CRISPR has made another case for the importance of basic research in the field of virus–host interactions. Contrary to the thought that most important questions about bacteria and bacteriophages have already been answered, there remains a huge gap in our knowledge about their interplay. The CRISPR/Cas system is part of an adaptive immunity in bacteria to protect against invading phages. In this system, two small RNAs are used to guide the Cas nuclease and cleave the invading nucleic acids in a sequence-specific manner. Targeted genome editing based on one particular CRISPR/Cas system known as CRISPR/Cas9 has emerged as a powerful and versatile tool for genome engineering in mammalian cells [15,16]. CRISPR/Cas9 editing has been used successfully to knock-out target gene expression in cells and animals. Compared to RNAi knockdown, the advantages of CRISPR/Cas9 knockout include ease, rapidity and robustness. It has therefore become the new standard for loss-of-function studies. In addition, other CRISPR-based technologies, including CRISPRa, CRISPRi and CRISPR screens, also hold a promise of substantially changing the study of viral pathogenesis and virus–host interactions, as reviewed by Gewurz and coworkers [2]. In short, CRISPR is game-changing and has tremendously advanced precision genome engineering. The applications of CRISPR technology will create an enormous impact on virology.

To push the applications of CRISPR technology in virology to the next stage, a multidisciplinary effort is required. Better communication and collaboration between bacteriologists, virologists and molecular biologists may bring about new innovations and out-of-box solutions to many important questions. Currently, only Cas9 has found wide applications in the precision editing of viral and cellular genomes. However, many other Cas nucleases have the potential to be used for different purposes in virology and antiviral therapy. Particularly, Cas3, which triggers RNA-guided DNA degradation other than single-site cleavage, seems to be the ideal enzyme for the development of antivirals against DNA viruses [17,18]. On the other hand, Cas13 and other nucleases that cleave RNA might prove useful in the fight against RNA viruses, as reviewed by Mahfouz et al. [13]. It will be of interest to see whether and how their use in plant viruses might currently be extended to pathogenic viruses of animals and humans. More basic research might be needed before these enzymes can be fully developed as a new tool in antiviral therapy. These and other exciting projects will keep us busy in the next few years.
